# Decisive Interactions between the Heterocyclic Moiety and the Cluster Observed in Polyoxometalate-Surfactant Hybrid Crystals

**DOI:** 10.3390/ijms16048505

**Published:** 2015-04-16

**Authors:** Saki Otobe, Natsumi Fujioka, Takuro Hirano, Eri Ishikawa, Haruo Naruke, Katsuhiko Fujio, Takeru Ito

**Affiliations:** 1 Department of Chemistry, School of Science, Tokai University, 4-1-1 Kitakaname, Hiratsuka 259-1292, Japan; E-Mails: sob0406@yahoo.co.jp (S.O.); sttok413@gmail.com (N.F.); tok413_st@yahoo.co.jp (T.H.); kfujio@tokai-u.jp (K.F.); 2 Department of Applied Chemistry, College of Engineering, Chubu University, 1200 Matsumoto, Kasugai, Aichi 487-8501, Japan; E-Mail: eishikawa@isc.chubu.ac.jp; 3 Chemical Resources Laboratory, Tokyo Institute of Technology, 4259-R1-23, Nagatsuta, Midori-ku, Yokohama 226-8503, Japan; E-Mail: hnaruke@gmail.com

**Keywords:** inorganic-organic, polyoxometalate, surfactant, heterocyclic, hybrid crystal

## Abstract

Inorganic-organic hybrid crystals were successfully obtained as single crystals by using polyoxotungstate anion and cationic dodecylpyridazinium (C_12_pda) and dodecylpyridinium (C_12_py) surfactants. The decatungstate (W_10_) anion was used as the inorganic component, and the crystal structures were compared. In the crystal comprising C_12_pda (C_12_pda-W_10_), the heterocyclic moiety directly interacted with W_10_, which contributed to a build-up of the crystal structure. On the other hand, the crystal consisting of C_12_py (C_12_py-W_10_) had similar crystal packing and molecular arrangement to those in the W_10_ crystal hybridized with other pyridinium surfactants. These results indicate the significance of the heterocyclic moiety of the surfactant to construct hybrid crystals with polyoxometalate anions.

## 1. Introduction

Weak chemical interactions, such as hydrogen bonding or van der Waals interactions, are crucial for the construction and function of biological molecules, such as proteins, DNA or RNA [1]. Employing these weak chemical interactions also provides effective options for building up synthetic molecular architectures [2,3,4,5]. To build up such molecular architectures, organic molecules or ligands are often used due to synthetic flexibility to control the directions and intensity of the weak chemical interactions. However, all-organic molecular architectures are less stable in the intermediate temperature (>100 °C) regions.

Inorganic-organic hybrid materials are more structurally stable than purely organic compounds owing to inorganic components, and the synergy of inorganic and organic characteristics will benefit constructing functional materials [6]. Conductive hybrid compounds composed of organic cations and inorganic anions have been reported, where the emergence of conductive functions is prompted by precise control of the molecular structures and arrangements of the components [7]. The precisely controlled inorganic-organic materials have been obtained as crystalline materials. 

As a molecular inorganic component, polyoxometalates (POMs) are promising candidates with respect to their structural and functional controllability [8,9,10,11,12,13,14,15,16]. POMs with various physicochemical properties have been successfully hybridized by structure-directing surfactants [17,18,19] to construct inorganic-organic crystalline hybrids [20,21,22,23,24] and single crystals [25,26,27,28,29,30,31,32,33,34]. Among several POM-surfactant single crystals, utilizing surfactants with a heterocyclic moiety enables the precise control of the composition and structure [31,32,33]. However, the variation of the surfactants has been limited to pyridinium and imidazolium cations.

Here, we report the syntheses and structures of polyoxotungstate hybrid crystals containing heterocyclic surfactants, including the first example of a POM-surfactant crystal comprised of a pyridazinium surfactant. The decatungstate ([W_10_O_32_]^4−^, W_10_) anion was hybridized with dodecylpyridazinium ([C_4_H_4_N_2_(C_12_H_25_)]^+^, C_12_pda) and dodecylpyridinium ([C_5_H_5_N(C_12_H_25_)]^+^, C_12_py) to form the crystals of C_12_pda-W_10_ (**1**) and C_12_py-W_10_ (**2**), respectively, and their chemical interactions and molecular arrangements were compared by X-ray structure analyses.

## 2. Results and Discussion

### 2.1. Crystal Structure of C_12_pda-W_10_ (**1**)

**Figure 1:**
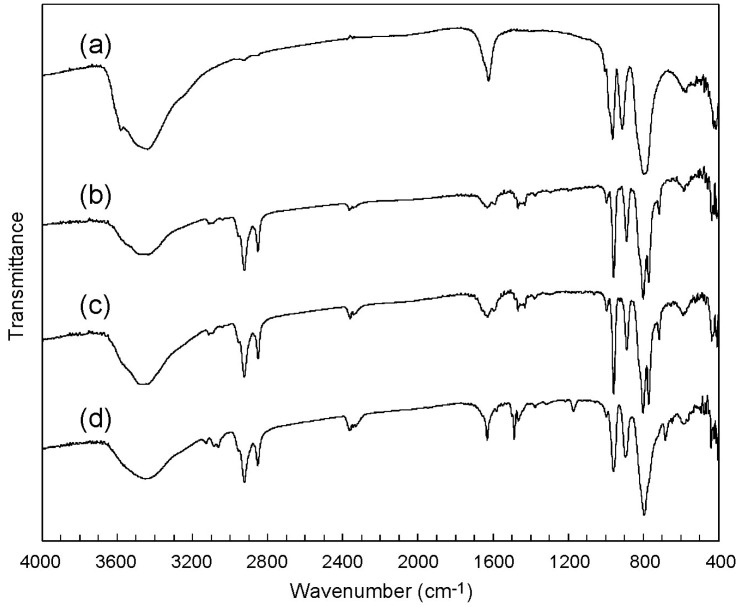
IR spectra of W_10_ compounds. (a) Na-W_10_ as a starting material; (b) As-prepared sample of **1**; (c) **1** after recrystallization; (d) As-prepared sample of **2**.

C_12_pda-W_10_ (**1**) was obtained by the cation exchange reaction of sodium salt of the W_10_ anion (Na-W_10_). The retention of the W_10_ structure before and after the recrystallization was confirmed by infrared (IR) spectra, which exhibited the characteristic peaks in the range of 400–1000 cm^−1^ (Figure 1a–c). Suitable single crystals for X-ray crystallography were obtained by employing acetone as the crystallization solvent.

**Figure 2:**
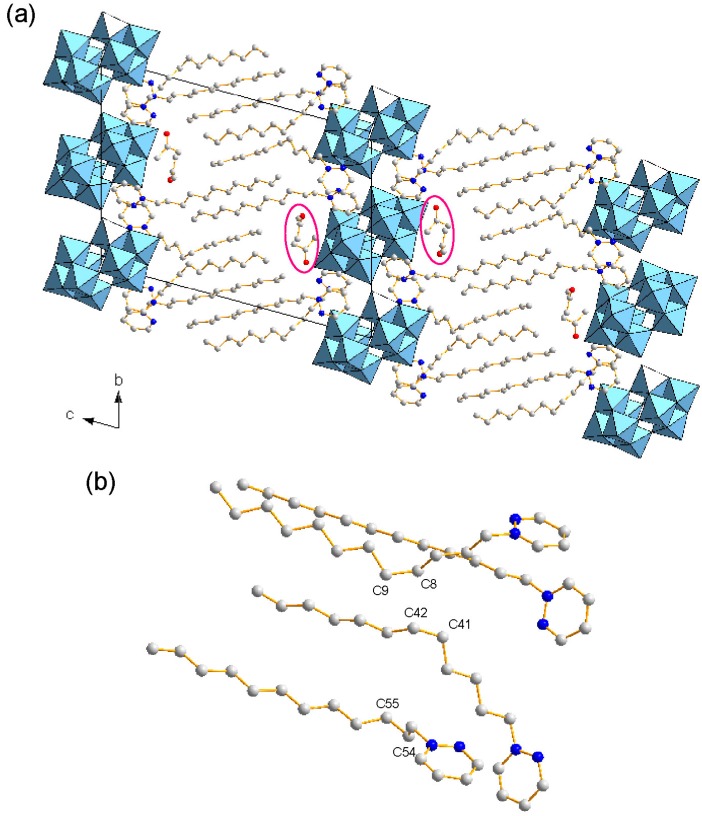
Crystal structure of **1** (C: gray, N: blue; W_10_ anions in polyhedral representations. H atoms are omitted for clarity). (**a**) Packing diagram along the *a* axis. Some acetone molecules are highlighted; (**b**) View of crystallographically-independent surfactant molecules.

The X-ray structure and elemental analyses revealed the formula of **1** to be [C_4_H_4_N_2_(C_12_H_25_)]_4_[W_10_O_32_]·2(CH_3_)_2_CO (Table 1). Four C_12_pda cations (1+ charge) were associated with one W_10_ anion (4− charge) due to the charge compensation. Figure 2 shows the crystal structure of **1**. The crystal packing consisted of alternating W_10_ inorganic monolayers and C_12_pda organic bilayers with a periodicity of 24.5 Å (Figure 2a). The acetone molecules were placed at the interface between the W_10_ and C_12_pda layers, being excluded from the inorganic layers. Although most C-C bonds of the dodecyl chains of C_12_pda had the anti conformation, three C-C bonds (C8-C9, C41-C42, C54-C55) had the gauche conformation (Figure 2b), two of which (C8-C9, C41-C42) were located at some methylene groups far away from the hydrophilic head.

**Table 1:** Crystallographic data.

Compound	1	2
Chemical formula	C_70_H_128_N_8_W_10_O_34_	C_76_H_140_N_4_W_10_O_36_
Formula weight	3464.31	3524.45
Crystal system	triclinic	triclinic
Space group	*P*$\bar{1}$ (No.2)	*P*$\bar{1}$ (No.2)
*a* (Å)	10.55918(19)	10.813(7)
*b* (Å)	18.7700(3)	11.339(7)
*c* (Å)	25.4318(5)	23.610(13)
α (°)	74.4842(7)	99.415(9)
β (°)	86.5737(7)	91.558(5)
γ (°)	85.6363(7)	115.588(9)
*V* (Å^3^)	4838.62(15)	2560(3)
*Z*	2	1
ρ_calcd_ (g·cm^−3^)	2.378	2.286
*T* (K)	193	173
μ (Mo·Kα) (mm^−1^)	11.924	11.272
No. of reflections measured	78,035	18,275
No. of independent reflections	22,146	11,774
*R*_int_	0.0900	0.1384
No. of parameters	1106	563
*R*_1_ (*I* > 2σ(*I*))	0.0452	0.0983
*wR*_2_ (all data)	0.1162	0.3237

**Figure 3:**
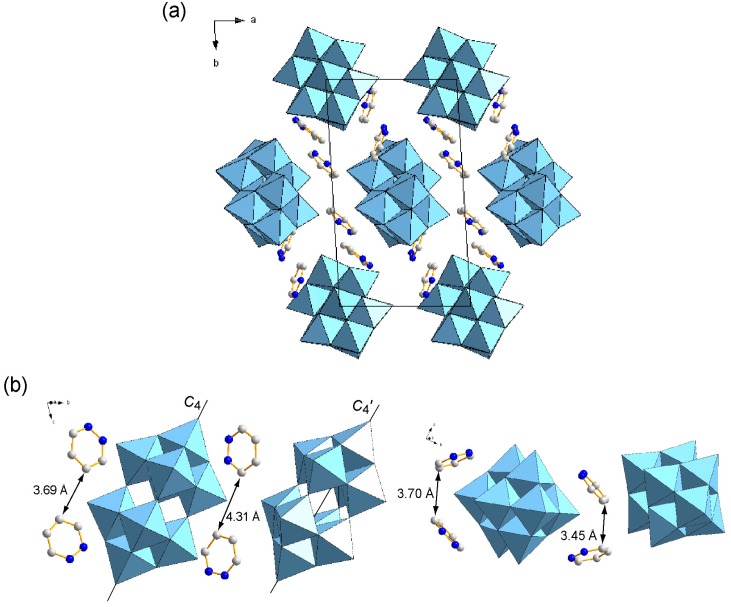
Molecular arrangements in the inorganic layers of **1** (C: gray, N: blue; W_10_ anions in polyhedral representations. H atoms and the dodecyl groups are omitted for clarity). (**a**) Packing diagram along the *c* axis; (**b**) View of crystallographically-independent pyridazinium moieties of surfactants in the vicinity of the W_10_ anions.

The hydrophilic heads of C_12_pda penetrated into the W_10_ inorganic layers and isolated each W_10_ anion (Figure 3a) in a similar way to that in the crystal of hexadecylpyridinium ([C_5_H_5_N(C_16_H_33_)]^+^, C_16_py) and W_10_ (C_16_py-W_10_, **3**) [32]. However, the conformations of the heterocyclic moiety were different. In **1**, the pyridazine rings of the C_12_pda cations were not in the vicinity of each other (Figure 3b), as in the case of the POM crystal comprising the pyridazinium cation without a long alkyl chain [35]. This indicates that there were no interactions, such as π–π stacking or the C-H···π interaction, between the heterocyclic moiety, being different from **2** (see below) and **3**. On the other hand, the pyridazine rings of C_12_pda interacted rather more directly with the W_10_ anions. The crystals of **1** had several short contacts between of O atoms of W_10_ and C or N atoms of the pyridazine ring (2.88–3.22 Å (mean: 3.09 Å); Table 2), indicating the direct interactions between W_10_ and the heterocyclic moiety of C_12_pda. The alignment of two crystallographically-independent W_10_ anions was not parallel, and the angle between the molecular *C*_4_ axes was 8.3° (Figure 3b).

**Table 2:** Short contacts between W_10_ and the heterocyclic moiety of C_12_pda in **1**.

Contact ^a^	Distance (Å)	Contact ^a^	Distance (Å)
C20^i^···O1	3.206	C2···O13	3.118
C20^i^···O2	3.085	C51^iv^···O13	3.201
C4···O5	3.210	C33^i^···O20	3.177
C19^i^···O5	3.147	C49···O22	2.914
C33^ii^···O6	3.140	C4···O24	2.886
C34^ii^···O6	3.180	C17···O25	2.894
C50^iii^···O7	2.876	C3^ii^···O27	3.216
C51^iii^···O7	3.110	C19^ii^···O27	3.163
C34^iii^···O8	3.134	C33^i^···O27	3.142
C4···O9	3.183	C17^i^···O28	3.135
C3···O9	2.890	C20···O29	3.119
N8^iii^···O12	3.046	N6···O30	2.909

^a^ Contact between O atoms of W_10_ and C or N atoms of the pyridazine ring of C_12_pda. Symmetry codes: (i) 1 + *x*, *y*, *z*; (ii) −*x*, −*y*, 2 − *z*; (iii) 1+*x*, −1 + *y*, *z*; (iv) *x*, −1 + *y*, *z*.

In the crystal of **1**, The C_12_pda cations had weak C-H···O hydrogen bonds [1]. Some C-H···O bonds were present in the vicinity of the gauche C-C bonds. The C···O distances were in the range of 2.89–3.74 Å (mean value: 3.32 Å), being much shorter than those of **2** (see below). In addition, most C-H···O hydrogen bonds were formed between W_10_ and the hydrophilic head of C_12_pda, *i.e.*, the pyridazine ring of C_12_pda. This suggests stronger interactions between W_10_ and the heterocyclic moiety of the surfactant in **1** than in **2**. These hydrogen bonds, as well as the electrostatic interaction between W_10_ and the heterocyclic moiety of C_12_pda stabilized the layered crystal structure of C_12_pda-W_10_ with rigid packing.

### 2.2. Crystal Structure of C_12_py-W_10_ (**2**)

C_12_py-W_10_ (**2**) was also synthesized by the cation exchange reaction of Na-W_10_ (Figure 1d). The molecular structure of W_10_ was retained before and after the recrystallization, as in the case of C_16_py-W_10_ (**3**) [32]. Although the recrystallization of **2** was difficult, some crystals obtained from ethanol were able to be analyzed by X-ray crystallography.

**Figure 4:**
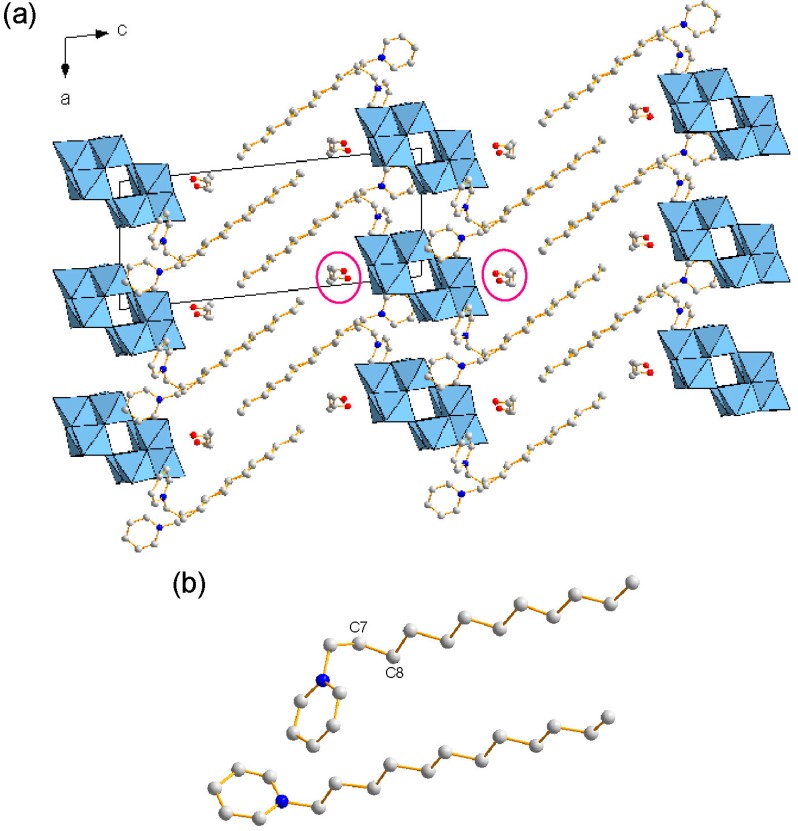
Crystal structure of **2** (C: gray, N: blue; W_10_ anions in polyhedral representations. H atoms are omitted for clarity). (**a**) Packing diagram along the *b* axis. Some ethanol molecules are highlighted; (**b**) View of crystallographically-independent surfactant molecules in **2**.

The formula of **2** was revealed to be [C_5_H_5_N(C_12_H_25_)]_4_[W_10_O_32_]·4C_2_H_5_OH (Table 1). Four C_12_py cations (1+ charge) were associated with one W_10_ anion (4− charge), being similar to **1** and **3**. The crystal packing of **2** consisted of alternating W_10_ inorganic monolayers and C_12_py organic bilayers with a distance of 23.2 Å (Figure 4a). Surprisingly, the cell parameters and the layered distances of **2** and **3** [32] were quite similar, even if the length of the pyridinium surfactants were changed to C_12_py (**2**) from C_16_py (**3**). The vacant spaces produced by changing to the shorter surfactant for **2** were filled by the ethanol molecules located at the interface between the W_10_ and C_12_py layers. This similarity of the layered structures led to the similar molecular arrangements of W_10_ and the pyridine rings (see below). All C-C bonds in the dodecyl chains showed the anti conformation except one C-C bond (C7-C8) near the hydrophilic head of C_12_py, being similar to that in **3** (Figure 4b).

**Figure 5:**
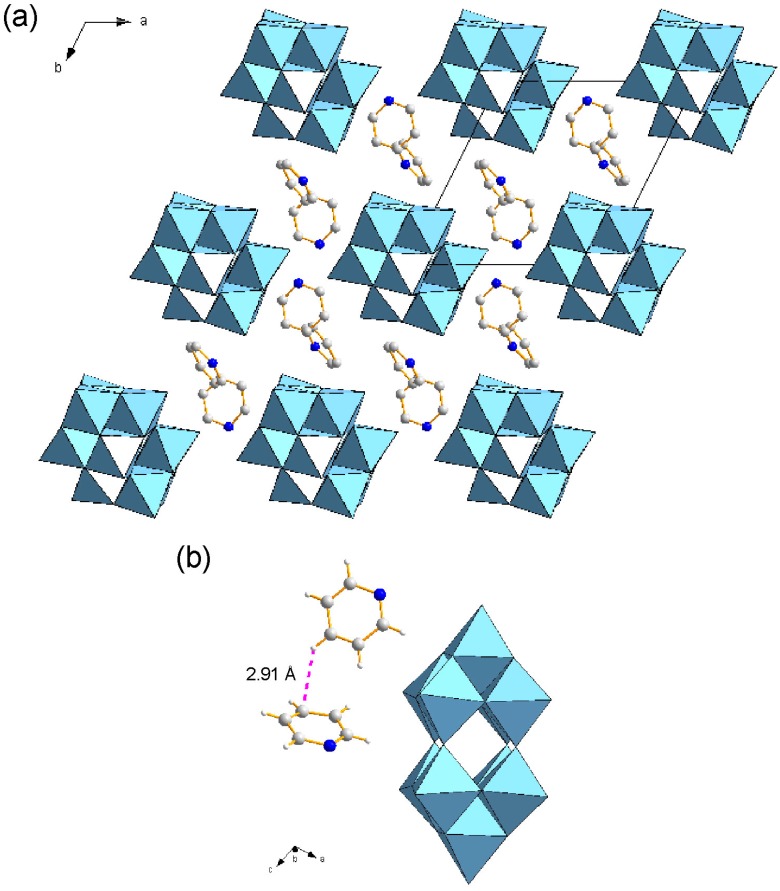
Molecular arrangements in the inorganic layers of **2** (C: gray, N: blue, H: white; W_10_ anions in polyhedral representations. The dodecyl groups are omitted for clarity). (**a**) Packing diagram along the *c* axis. H atoms are omitted for clarity; (**b**) View of crystallographically-independent pyridinium moieties of surfactants in the vicinity of the W_10_ anions. The selected short contact is presented as a pink dotted line.

The hydrophilic heads of C_12_py penetrated into the W_10_ inorganic layers in **2** (Figure 5a), which was almost the same manner as observed in **3**. The alignment of each W_10_ anion was parallel, being similar to **3**, but different from **1**. The penetrated C_12_py cations formed two crystallographically-independent pairs (Figure 5b). The two C_12_py cations interacted weakly through the C-H···π interaction, where the shortest interatomic distance was 2.91 Ǻ for C3···H20 (Figure 5b). In the crystal of **2**, there were short contacts between W_10_ and the pyridine ring (2.93–3.22 Å (mean: 3.07 Å), Table 3). **2** also had C-H···O hydrogen bonds at the interface between the W_10_ and C_12_py layers (C···O distance: 3.03–3.88 Å; mean value: 3.52 Å), being much longer than those observed in **1**. These longer C-H···O hydrogen bonds were formed around W_10_ and the pyridine ring of C_12_py, indicating that the interactions between W_10_ and the heterocyclic moiety in **2** were weaker than in **1**. The structure of the heterocyclic moiety in the surfactants is the significant factor to construct the POM-surfactant hybrid crystals.

**Table 3:** Short contacts between W_10_ and the heterocyclic moiety of C_12_py in **2**.

Contact ^a^	Distance (Å)	Contact ^a^	Distance (Å)
C22···O5	3.153	C20^ii^···O11	3.026
N2^i^···O9	2.929	N1^iii^···O14	2.960
C18^i^···O9	2.980	C5^iii^···O14	3.203
C22^i^···O9	3.108	C21^iv^···O14	3.091
C2···O11	3.220	C18^v^···O16	3.031

^a^ Contact between O atoms of W_10_ and C or N atoms of the pyridine ring of C_12_py. Symmetry codes: (i) −1 + *x*, *y*, *z*; (ii) 1 − *x*, 1 − *y*, −*z*; (iii) *x*, −1 + *y*, *z*; (iv) 1 − *x*, −*y*, −*z*; (v) −1 + *x*, −1 + *y*, *z*.

## 3. Experimental Section

### 3.1. Syntheses and Methods

All chemical reagents were obtained from commercial sources (Wako, Osaka, Japan). [C_4_H_4_N_2_(C_12_H_25_)]Br (C_12_pda·Br) was synthesized by using pyridazine and 1-bromododecane based on the literature [36]. Na_4_[W_10_O_32_] (Na-W_10_) was synthesized by combining a boiled solution of Na_2_WO_4_·2H_2_O (0.5 M, 25 mL) and boiled HCl (1 M, 25 mL) [37]. 

C_12_pda-W_10_ (**1**) was synthesized by the cation exchange of Na-W_10_. Na-W_10_ (0.50 g, 0.20 mmol) was dissolved in 20 mL of HCl (pH = 2), and ethanol solution containing 0.27 g (0.81 mmol) of C_12_pda·Br was added. After stirring for 10 min, the obtained white precipitates were filtered and dried. Recrystallization of the crude product from hot acetone gave colorless plates of **1**. The crystals of **1** were efflorescent, and its elemental composition was calculated for the formula without the solvent of crystallization. Data for **1**: anal. calcd. for C_64_H_116_N_8_W_10_O_32_: C, 22.96; H, 3.49; N, 3.35%; found: C, 22.77; H, 3.36; N, 3.33%. IR (KBr disk): 996 (w), 958 (s), 889 (m), 800 (s), 772 (s), 589 (w), 437 (m), 407 (m) cm^−1^.

C_12_py-W_10_ (**2**) was synthesized by a similar procedure as for **1**. [C_5_H_5_N(C_12_H_25_)]Br (C_12_py·Br, 0.28 g (0.81 mmol)) was employed as the cationic surfactant. The crude product was recrystallized from ethanol to obtain colorless prisms of **2**, which were efflorescent. Data for **1**: anal. calcd. for C_76_H_120_N_4_W_10_O_32_: C, 24.42; H, 3.62; N, 1.68%; found: C, 24.01; H, 3.42; N, 1.62%. IR (KBr disk): 997 (w), 959 (s), 895 (m), 796 (s), 683 (m), 584 (w), 440 (m), 410 (m) cm^−1^.

### 3.2. X-ray Diffraction Measurements

Single-crystal X-ray diffraction measurements for **1** were made on a Rigaku RAXIS RAPID imaging plate diffractometer with graphite monochromated Mo-Kα radiation (λ = 0.71075 Å). Diffraction data were collected and processed with PROCESS-AUTO [38]. The structure of **1** was solved by the charge flipping method [39] and expanded using Fourier techniques. The refinement procedure was performed by the full-matrix least-squares using SHELXL97 [40]. The measurements for **2** were made on a Rigaku Saturn70 diffractometer using graphite monochromated Mo-Kα radiation. Diffraction data were collected and processed with CrystalClear [41]. The structure of **2** was solved by direct methods [42] and expanded using Fourier techniques. The refinement procedure was performed by full-matrix least-squares using SHELXL2013 [42]. All calculations were performed using the CrystalStructure [43] software package. In the refinement procedure, all non-hydrogen atoms, except for C37 of **2**, were refined anisotropically, and the hydrogen atoms on C atoms were located in the calculated positions. The weak reflection intensities from the crystals of **2** may result in relatively high *R*_1_ and *wR*_2_ values. The results of checking cif files are available as supplementary materials. Further details of the crystal structure investigation may be obtained free of charge from the Cambridge Crystallographic Data Centre, 12 Union Road, Cambridge CB2 1EZ, UK; Fax: +44 1223 336 033; or E-Mail: deposit@ccdc.cam.ac.uk (CCDC 1055676-1055677).

## 4. Conclusions

Inorganic-organic hybrid crystals comprised of polyoxotungstate and surfactants having a heterocyclic moiety, [C_4_H_4_N_2_(C_12_H_25_)]_4_[W_10_O_32_]·2(CH_3_)_2_CO (**1**) and [C_5_H_5_N(C_12_H_25_)]_4_[W_10_O_32_]·4C_2_H_5_OH (**2**), were successfully synthesized. Dodecylpyridazinium (C_12_pda) and dodecylpyridinium (C_12_py) were utilized as organic cations, and the decatungstate anion (W_10_) was used for the inorganic component, which enabled the discussion of the effect of the heterocyclic moiety for the construction of the hybrid crystals. Although both hybrid crystals contained alternate stacking of W_10_ monolayers and interdigitated surfactant bilayers, pyridazine rings in **1** interacted more strongly with the W_10_ anions. **1** contained shorter C-H···O hydrogen bonds than those observed in **2**, indicating that these hydrogen bonds, as well as the electrostatic interaction between the C_12_pda cation and the W_10_ anion stabilized the layered structure of C_12_pda-W_10_ with rigid packing. On the other hand, **2** has similar cell parameters and molecular arrangements as those in the crystals of hexadecylpyridinium (C_16_py) and W_10_ (**3**), also indicating the significance of the heterocyclic moiety for the construction of the hybrid crystals. The difference between the heterocyclic moieties led to different arrangements of W_10_ anions in **1** and **2**, which will contribute to the precise control of molecular arrangements and the emergence of characteristic conductivity.

## Supplementary Materials

Supplementary materials can be found at http://www.mdpi.com/1422-0067/16/04/8505/s1.
