# Supplementary material for: Decisive Interactions between the Heterocyclic Moiety and the Cluster Observed in Polyoxometalate-Surfactant Hybrid Crystals
Source: Int J Mol Sci. 2015 Apr 16;16(4):8505–16. doi: 10.3390/ijms16048505 (PMC4425093; doi:10.3390/ijms16048505)
Supplement: Supplementary file 1 [file ijms-16-08505-s001.pdf]

Supplementary Information

Check Report of cif for 1 (C_{12}pda-W_{10})

CheckCIF/PLATON Report

You have not supplied any structure factors. As a result the full set of tests cannot be run.
THIS REPORT IS FOR GUIDANCE ONLY. IF USED AS PART OF A REVIEW PROCEDURE FOR PUBLICATION, IT SHOULD NOT REPLACE THE EXPERTISE OF AN EXPERIENCED CRYSTALLOGRAPHIC REFEREE.
No syntax errors found. CIF dictionary Interpreting this report

Datablock: 140304

```
Bond precision:  C-C = 0.0183 A          Wavelength=0.71075

Cell:           a=10.55918(19)     b=18.7700(3)      c=25.4318(5)
                alpha=74.4842(7)   beta=86.5737(7)   gamma=85.6363(7)
Temperature:    193 K
```

	Calculated	Reported
Volume	4838.63(15)	4838.62(15)
Space group	P -1	P -1
Hall group	-P 1	-P 1
Moiety formula	O32 W10, 4(C16 H29 N2), 2(C3 H6 O)	C70 H128 N8 O34 W10
Sum formula	C70 H128 N8 O34 W10	C70 H128 N8 O34 W10
Mr	3464.20	3464.31
Dx,g cm-3	2.378	2.378
Z	2	2
Mu (mm-1)	11.910	11.924
F000	3232.0	3232.0
F000’	3218.05	
h,k,lmax	13,24,33	13,24,33
Nref	22200	22146
Tmin,Tmax	0.249,0.551	0.139,0.551
Tmin’	0.001	

```
Correction method= MULTI-SCAN

Data completeness= 0.998          Theta(max)= 27.490

R(reflections)= 0.0452( 17651)    wR2(reflections)= 0.1162( 22146)

S = 1.034                  Npar= 1106
```

The following ALERTS were generated. Each ALERT has the format

test-name_ALERT_alert-type_alert-level

Click on the hyperlinks for more details of the test.

Alert level C

```
PLAT220_ALERT_2_C Large Non-Solvent   C     Ueq(max)/Ueq(min) Range        3.9 Ratio
PLAT230_ALERT_2_C Hirshfeld Test Diff for    N6     --  C33     ..        5.2 su
PLAT234_ALERT_4_C  Large  Hirshfeld  Difference  N5    --  N6      ..       0.19 Ang.
PLAT234_ALERT_4_C  Large  Hirshfeld  Difference  N7    --  N8      ..       0.18 Ang.
PLAT234_ALERT_4_C  Large  Hirshfeld  Difference  N7    --  C52     ..       16.  Ang.
PLAT234_ALERT_4_C  Large  Hirshfeld  Difference  N8    --  C49     ..       17.  Ang.
PLAT234_ALERT_4_C Large Hirshfeld Difference C51     --  C52     ..       0.20 Ang.
PLAT241_ALERT_2_C High       Ueq  as  Compared  to  Neighbors  for  .....     N6 Check
PLAT241_ALERT_2_C High       Ueq  as  Compared  to  Neighbors  for  .....     C47 Check
PLAT241_ALERT_2_C High       Ueq  as  Compared  to  Neighbors  for  .....     N8 Check
PLAT241_ALERT_2_C High       Ueq  as  Compared  to  Neighbors  for  .....     C55 Check
PLAT242_ALERT_2_C Low        Ueq  as  Compared  to  Neighbors  for  .....     C33 Check
PLAT242_ALERT_2_C Low        Ueq as Compared to Neighbors for .....      C46 Check
PLAT244_ALERT_4_C Low    'Solvent' Ueq as Compared to Neighbors of         C66 Check
PLAT342_ALERT_3_C Low Bond Precision on   C-C Bonds ...............     0.0183 Ang.
PLAT360_ALERT_2_C Short   C(sp3)-C(sp3)  Bond   C44     -   C45    ...      1.42 Ang.
PLAT362_ALERT_2_C Short   C(sp3)-C(sp2)  Bond   C66     -   C67    ...      1.40 Ang.
PLAT790_ALERT_4_C Centre of Gravity not Within Unit Cell: Resd.    #          1 Note
              O32 W10
```

Alert level G

```
CHEMS02_ALERT_1_G  Please check that you have entered the correct
            _publ_requested_category classification of your compound;
            FI or CI or EI for inorganic; FM or CM or EM for metal-organic;
            FO or CO or EO for organic.
            From the CIF: _publ_requested_category    CHOOSE FI FM FO CI CM CO or
            From the CIF: _chemical_formula_sum:C70 H128 N8 O34 W10
PLAT005_ALERT_5_G No _iucr_refine_instructions_details   in the CIF      Please Do !
PLAT042_ALERT_1_G Calc. and Reported MoietyFormula Strings   Differ      Please Check
PLAT083_ALERT_2_G SHELXL Second Parameter in WGHT Unusually Large.        23.11 Why ?
PLAT154_ALERT_1_G The su's on the Cell Angles are Equal .........     0.00070 Degree
PLAT380_ALERT_4_G Incorrectly? Oriented X(sp2)-Methyl Moiety .....         C67 Check
PLAT432_ALERT_2_G Short  Inter  X...Y  Contact  O7      ..  C50      ..       88.  Ang.
PLAT432_ALERT_2_G Short  Inter  X...Y  Contact  O9      ..  C3       ..       89.  Ang.
PLAT432_ALERT_2_G Short  Inter  X...Y  Contact  O22     ..  C49      ..       2.91 Ang.
PLAT432_ALERT_2_G Short  Inter  X...Y  Contact  O24     ..  C4       ..       2.89 Ang.
PLAT432_ALERT_2_G Short Inter X...Y Contact  O25     ..  C17      ..       2.89 Ang.
PLAT790_ALERT_4_G Centre of Gravity not Within Unit Cell: Resd.    #          5 Note
              C16 H29 N2
PLAT790_ALERT_4_G Centre of Gravity not Within Unit Cell: Resd.    #          8 Note
              C3 H6 O
```

```
   0 ALERT level A = Most likely a serious problem - resolve or explain
   0 ALERT level B = A potentially serious problem, consider carefully
  18 ALERT level C = Check. Ensure it is not caused by an omission or oversight
  13 ALERT level G = General information/check it is not something unexpected

   3 ALERT type 1 CIF construction/syntax error, inconsistent or missing data
  16 ALERT type 2 Indicator that the structure model may be wrong or deficient
   1 ALERT type 3 Indicator that the structure quality may be low
  10 ALERT type 4 Improvement, methodology, query or suggestion
   1 ALERT type 5 Informative message, check
```

Check report of cif for 2 (C_{12}py-W_{10})

CheckCIF/PLATON Report

You have not supplied any structure factors. As a result the full set of tests cannot be run.
THIS REPORT IS FOR GUIDANCE ONLY. IF USED AS PART OF A REVIEW PROCEDURE FOR PUBLICATION, IT SHOULD NOT REPLACE THE EXPERTISE OF AN EXPERIENCED CRYSTALLOGRAPHIC REFEREE.
No syntax errors found. CIF dictionary Interpreting this report

Datablock: TOtobeSaturn4

```
Bond precision:  C-C = 0.0419 A          Wavelength=0.71075

Cell:            a=10.813(7)       b=11.339(7)      c=23.610(13)
                 alpha=99.415(9)   beta=91.558(5)   gamma=115.588(9)
Temperature:     173 K
```

	Calculated	Reported
Volume	2560(3)	2560(3)
Space group	P -1	P -1
Hall group	-P 1	-P 1
Moiety formula	O32 W10, 4(C17 H30 N), 4(C2 H6 O)	C76 H140 N4 O36 W10
Sum formula	C76 H144 N4 O36 W10	C76 H140 N4 O36 W10
Mr	3528.35	3524.45
Dx,g cm-3	2.289	2.286
Z	1	1
Mu (mm-1)	11.258	11.272
F000	1656.0	1652.0
F000'	1649.02	
h,k,lmax	15,16,34	15,16,34
Nref	17033	11774
Tmin,Tmax	0.131,0.105	0.046,0.105
Tmin'	0.084	

```
Correction method= MULTI-SCAN

Data completeness= 0.691          Theta(max)= 31.493

R(reflections)= 0.0983( 8235)     wR2(reflections)= 0.3237( 11774)

S = 1.058                  Npar= 563
```

The following ALERTS were generated. Each ALERT has the format
test-name_ALERT_alert-type_alert-level
Click on the hyperlinks for more details of the test.

Alert level A

```
PLAT029_ALERT_3_A _diffrn_measured_fraction_theta_full Low .......      0.812 Note
```

Author Response: ...This structure intrinsically exhibits many weak reflections at higher theta values. The submitted manuscript mainly reports the packing mode of the decatungstate anions and pyridinium cations. The data obtained had insufficient quality, but was sufficient to elucidate the packing feature of the decatungstate anion and heterocyclic moiety.

Alert level B

```
PLAT342_ALERT_3_B Low Bond Precision on   C-C Bonds ...............      0.0419 Ang.
```

Alert level C

```
DIFMN02_ALERT_2_C  The minimum difference density is < -0.1*ZMAX*0.75
            _refine_diff_density_min given =      -6.420
            Test value =      -5.550
DIFMN03_ALERT_1_C  The minimum difference density is < -0.1*ZMAX*0.75
            The relevant atom site should be identified.
RFACR01_ALERT_3_C  The value of the weighted R factor is > 0.25
            Weighted R factor given    0.324
RINTA01_ALERT_3_C  The value of Rint is greater than 0.12
            Rint given   0.138
PLAT020_ALERT_3_C The value of Rint is greater than 0.12 .........      0.138 Report
PLAT041_ALERT_1_C Calc. and Reported SumFormula     Strings  Differ     Please Check
PLAT043_ALERT_1_C Calculated and Reported Mol. Weight Differ by ..       3.90 Check
PLAT068_ALERT_1_C Reported F000 Differs from Calcd (or Missing)...     Please Check
PLAT084_ALERT_3_C High wR2 Value (i.e. > 0.25) ...................       0.32 Report
PLAT098_ALERT_2_C Large Reported Min.  (Negative) Residual Density      -6.42 eA-3
PLAT202_ALERT_3_C Isotropic non-H Atoms in Anion/Solvent .........          1
PLAT213_ALERT_2_C Atom O1                  has ADP max/min Ratio .....    3.1 oblate
PLAT213_ALERT_2_C Atom C1                  has ADP max/min Ratio .....    3.8 prolat
PLAT213_ALERT_2_C Atom C10                 has ADP max/min Ratio .....    3.7 prolat
PLAT213_ALERT_2_C Atom C14                 has ADP max/min Ratio .....    3.4 oblate
PLAT213_ALERT_2_C Atom C15                 has ADP max/min Ratio .....    3.4 prolat
PLAT213_ALERT_2_C Atom C18                 has ADP max/min Ratio .....    3.2 oblate
PLAT220_ALERT_2_C Large Non-Solvent   C      Ueq(max)/Ueq(min)  Range    3.1 Ratio
PLAT230_ALERT_2_C Hirshfeld Test Diff for      C1      --   C2      ..    6.0 su
PLAT234_ALERT_4_C Large Hirshfeld Difference W1      --   O1      ..   0.18 Ang.
PLAT234_ALERT_4_C Large Hirshfeld Difference W3      --   O11     ..   0.16 Ang.
PLAT234_ALERT_4_C Large Hirshfeld Difference C3      --   C4      ..   0.20 Ang.
PLAT234_ALERT_4_C Large Hirshfeld Difference C8      --   C9      ..   0.19 Ang.
PLAT241_ALERT_2_C High       Ueq as Compared to Neighbors for .....    C1 Check
PLAT242_ALERT_2_C Low        Ueq as Compared to Neighbors for .....    C2 Check
PLAT242_ALERT_2_C Low        Ueq as Compared to Neighbors for .....    C32 Check
PLAT244_ALERT_4_C Low    'Solvent' Ueq as Compared to Neighbors of    C35 Check
PLAT244_ALERT_4_C Low    'Solvent' Ueq as Compared to Neighbors of    C37 Check
PLAT250_ALERT_2_C Large U3/U1 Ratio for Average U(i,j) Tensor ....    2.4 Note
PLAT360_ALERT_2_C  Short  C(sp3)-C(sp3) Bond   C33    -   C34    ...   1.36 Ang.
PLAT360_ALERT_2_C  Short  C(sp3)-C(sp3) Bond   C37    -   C38    ...   1.38 Ang.
PLAT361_ALERT_2_C Long    C(sp3)-C(sp3) Bond  C29     -   C30    ...   1.67 Ang.
PLAT415_ALERT_2_C Short Inter D-H..H-X        H18E   ..   H23A    ..   2.10 Ang.
```

Alert level G

```
FORMU01_ALERT_2_G  There is a discrepancy between the atom counts in the
            _chemical_formula_sum and the formula from the _atom_site* data.
            Atom count from _chemical_formula_sum:C76 H140 N4 O36 W10
            Atom count from the _atom_site data:   C76 H144 N4 O36 W10
CELLZ01_ALERT_1_G Difference between formula and atom_site contents detected.
CELLZ01_ALERT_1_G ALERT: Large difference may be due to a
            symmetry error - see SYMMG tests
           From the CIF: _cell_formula_units_Z     1
           From the CIF: _chemical_formula_sum   C76 H140 N4 O36 W10
           TEST: Compare cell contents of formula and atom_site data

           atom    Z*formula  cif sites diff
           C         76.00     76.00    0.00
           H        140.00    144.00   -4.00
           N          4.00      4.00    0.00
           O         36.00     36.00    0.00
           W         10.00     10.00    0.00
CHEMS02_ALERT_1_G  Please check that you have entered the correct
            _publ_requested_category classification of your compound;
            FI or CI or EI for inorganic; FM or CM or EM for metal-organic;
            FO or CO or EO for organic.
            From the CIF: _publ_requested_category    CHOOSE FI FM FO CI CM CO or
            From the CIF: _chemical_formula_sum:C76 H140 N4 O36 W10
PLAT005_ALERT_5_G No _iucr_refine_instructions_details   in the CIF     Please Do !
PLAT007_ALERT_5_G Number of Unrefined Donor-H Atoms .............           2 Report
PLAT042_ALERT_1_G Calc. and Reported MoietyFormula Strings   Differ     Please Check
PLAT072_ALERT_2_G SHELXL First   Parameter in WGHT Unusually Large.       0.20 Report
PLAT432_ALERT_2_G Short Inter X...Y Contact   O9     ..  C18     ..       2.99 Ang.
```

```
  1 ALERT level A = Most likely a serious problem - resolve or explain
  1 ALERT level B = A potentially serious problem, consider carefully
 33 ALERT level C = Check. Ensure it is not caused by an omission or oversight
  9 ALERT level G = General information/check it is not something unexpected

  8 ALERT type 1 CIF construction/syntax error, inconsistent or missing data
 21 ALERT type 2 Indicator that the structure model may be wrong or deficient
  7 ALERT type 3 Indicator that the structure quality may be low
  6 ALERT type 4 Improvement, methodology, query or suggestion
  2 ALERT type 5 Informative message, check
```
